# Supplementary material for: Saccharomyces boulardii (CNCM I-745) improves intestinal damage in sepsis by remodeling intestinal flora structure
Source: Front Cell Infect Microbiol. 2025 Jul 31;15:1602792. doi: 10.3389/fcimb.2025.1602792 (PMC12350329; doi:10.3389/fcimb.2025.1602792)
Supplement: Supplementary file 1 [file DataSheet1.zip › summary/summary/8_Advanced_analyse/2_PICRUSt2_result/KEGG/Group.BW4_vs_BW0/PICRUSt2_KO_Group.BW4_vs_BW0_diff.pdf]

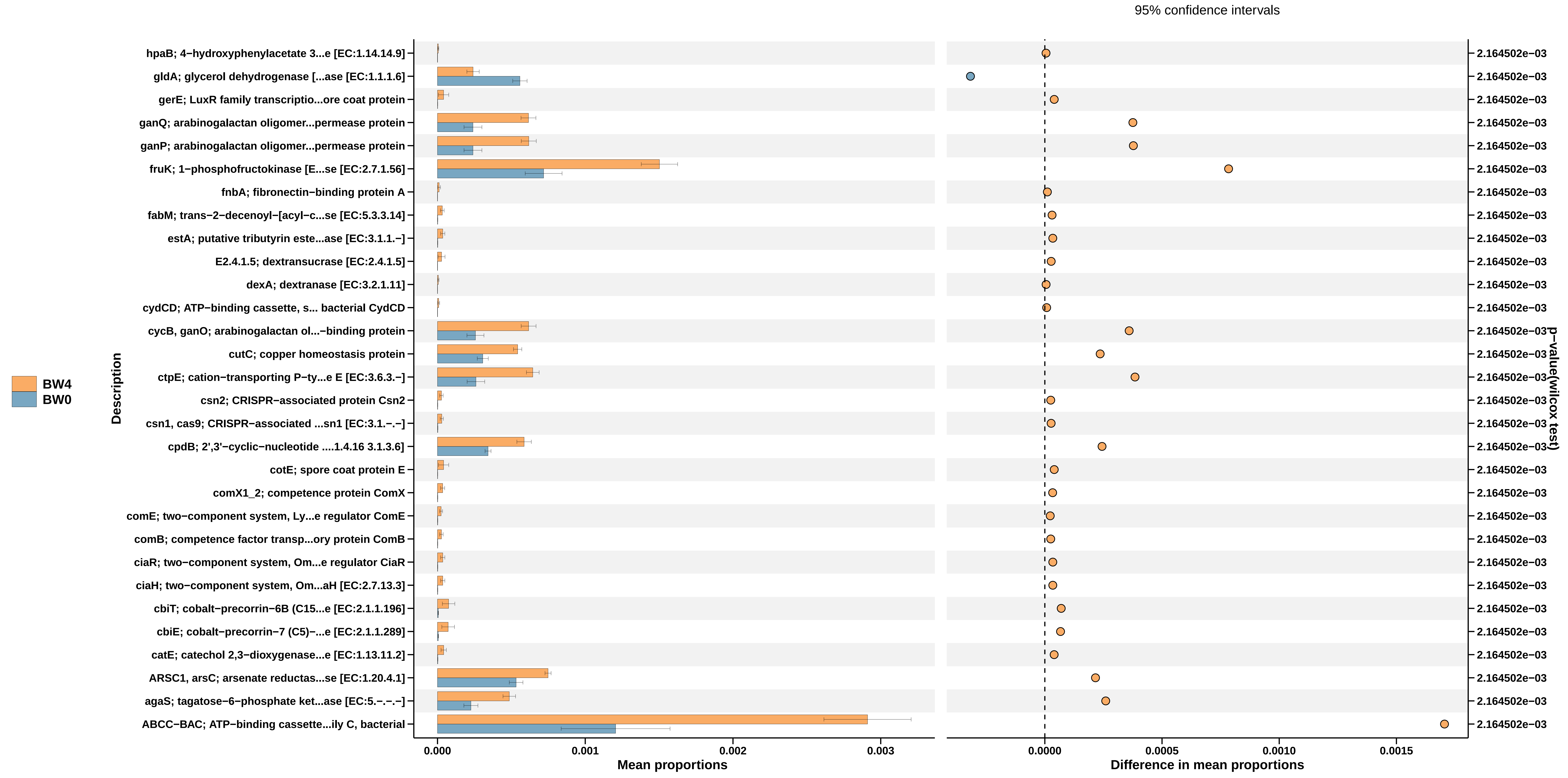

95% confidence intervals
hpaB; 4−hydroxyphenylacetate 3...e [EC:1.14.14.9]
gldA; glycerol dehydrogenase [...ase [EC:1.1.1.6]
gerE; LuxR family transcriptio...ore coat protein
ganQ; arabinogalactan oligomer...permease protein
ganP; arabinogalactan oligomer...permease protein
fruK; 1−phosphofructokinase [E...se [EC:2.7.1.56]
fnbA; fibronectin−binding protein A
fabM; trans−2−decenoyl−[acyl−c...se [EC:5.3.3.14]
estA; putative tributyrin este...ase [EC:3.1.1.−]
E2.4.1.5; dextransucrase [EC:2.4.1.5]
dexA; dextranase [EC:3.2.1.11]
cydCD; ATP−binding cassette, s... bacterial CydCD
cycB, ganO; arabinogalactan ol...−binding protein
cutC; copper homeostasis protein
ctpE; cation−transporting P−ty...e E [EC:3.6.3.−]
csn2; CRISPR−associated protein Csn2
csn1, cas9; CRISPR−associated ...sn1 [EC:3.1.−.−]
cpdB; 2',3'−cyclic−nucleotide ....1.4.16 3.1.3.6]
cotE; spore coat protein E
comX1_2; competence protein ComX
comE; two−component system, Ly...e regulator ComE
comB; competence factor transp...ory protein ComB
ciaR; two−component system, Om...e regulator CiaR
ciaH; two−component system, Om...aH [EC:2.7.13.3]
cbiT; cobalt−precorrin−6B (C15...e [EC:2.1.1.196]
cbiE; cobalt−precorrin−7 (C5)−...e [EC:2.1.1.289]
catE; catechol 2,3−dioxygenase...e [EC:1.13.11.2]
ARSC1, arsC; arsenate reductas...se [EC:1.20.4.1]
agaS; tagatose−6−phosphate ket...ase [EC:5.−.−.−]
ABCC−BAC; ATP−binding cassette...ily C, bacterial
Description
BW4
BW0
0.000
0.001
0.002
0.003
Mean proportions
0.0000
0.0005
0.0010
0.0015
Difference in mean proportions
p−value(wilcox test)
2.164502e−03
